# Beneficial Effect of Jojoba Seed Extracts on Hyperglycemia-Induced Oxidative Stress in RINm5f Beta Cells

**DOI:** 10.3390/nu10030384

**Published:** 2018-03-20

**Authors:** Sahla Belhadj, Olfa Hentati, Ghaith Hamdaoui, Khaskhoussi Fakhreddine, Elisa Maillard, Stéphanie Dal, Séverine Sigrist

**Affiliations:** 1 UMR DIATHEC, EA 7294, Fédération de Médecine Translationnelle de Strasbourg (FMTS), University of Strasbourg, Boulevard René Leriche, 67200 Strasbourg, France; belhadjsahla@hotmail.com (S.B.); e.pedracini@ceed-diabete.org (E.M.); dalstephaniepro67@gmail.com (S.D.); 2 Institut Supérieur de Biotechnologie de Sfax (ISBS), Road of Soukra Km 4, PO Box, Sfax 1175, Tunisia; olfa_hentati@yahoo.fr; 3 Laboratory of Bioactive Substances, Center of Biotechnology of Borj Cédria, BP 901, Hammam-lif 2050, Tunisia; ghaith_cbbc@yahoo.fr; 4 AGRO-CRC, Al Amine Al Abbassi Street, Tunis 1002, Tunisia; khaskhoussif@yahoo.fr

**Keywords:** RINm5f, oxidative stress, jojoba, caspase-3, Nrf2, p22phox

## Abstract

Hyperglycemia occurs during diabetes and insulin resistance. It causes oxidative stress by increasing reactive oxygen species (ROS) levels, leading to cellular damage. Polyphenols play a central role in defense against oxidative stress. In our study, we investigated the antioxidant properties of simmondsin, a pure molecule present in jojoba seeds, and of the aqueous extract of jojoba seeds on fructose-induced oxidative stress in RINm5f beta cells. The exposure of RINm5f beta cells to fructose triggered the loss of cell viability (−48%, *p* < 0.001) and disruption of insulin secretion (*p* < 0.001) associated with of reactive oxygen species (ROS) production and a modulation of pro-oxidant and antioxidant signaling pathway. Cell pre-treatments with extracts considerably increased cell viability (+86% *p* < 0.001) for simmondsin and +74% (*p* < 0.001) for aqueous extract and insulin secretion. The extracts also markedly decreased ROS (−69% (*p* < 0.001) for simmondsin and −59% (*p* < 0.001) for aqueous extract) and caspase-3 activation and improved antioxidant defense, inhibiting p22phox and increasing nuclear factor (erythroid-derived 2)-like 2 (Nrf2) levels (+70%, *p* < 0.001) for aqueous extract. Simmondsin had no impact on Nrf2 levels. The richness and diversity of molecules present in jojoba seed extract makes jojoba a powerful agent to prevent the destruction of RINm5f beta cells induced by hyperglycemia.

## 1. Introduction

Diabetes affects 415 million adults (8.5%) worldwide, and this figure is likely to increase to more than 642 million by 2040 [1]. Diabetes has become a major cause of death in people younger than 60 years [1]. This metabolic disorder is characterized by relative or absolute deficiency of insulin secretion and/or insulin resistance that cause chronic hyperglycemia and impaired carbohydrates, lipids, and proteins metabolism [2]. Two major types of diabetes are known, type 1 and type 2, characterized by insulin defects such as insulin deficiency and insulin resistance [3], respectively.

Obesity is a major risk factor for type 2 diabetes. The term “diabesity” clearly shows this association. Diabetes and obesity are linked to a chronic stress and redox equilibrium disorder that lead to numerous complications associated with micro and macro-angiopathies [4].

During diabetes or insulin resistance, failure of insulin-stimulated glucose uptake by fat and muscle causes high glucose concentration in blood. Consequently, glucose uptake by insulin-independent tissues, oxidant production, and impaired antioxidant defenses occur [5].

Oxidative stress is the result of an imbalance between pro-oxidant and antioxidant homeostasis in favor of pro-oxidant homeostasis, inducing the generation of toxic reactive oxygen species (ROS) such as organic hydroperoxides, hydrogen peroxide (H_2_O_2_), nitric oxide, superoxide, or hydroxyl radicals [6].

Oxidative stress is characterized by the high production of ROS and the decrease in antioxidant activity [7,8]. According to several investigations, reduced nicotinamide adenine dinucleotide phosphate (NADPH) oxidases are considered to be the key source of ROS [9,10]. The assembly of several subunits such as p67phox and p47phox with the cytochrome b558 bound to the membrane through subunits such as p22phox is required to activate the NADPH enzymatic complex [10]. On the other hand, the nuclear factor (erythroid-derived 2)-like 2 (Nrf2), a transcription factor located in the cytochrome, plays a key role in the defense against oxidative stress, reacting with Kelch-like erythroid-cell-derived protein with Cap ‘n’ Collar homology (ECH)-associated protein 1 (Keap-1) and getting easily degraded by the ubiquitin-proteasome [11]. During oxidative stress, Nrf2 is detached from Keap-1 and translocates to the nucleus to promote the expression of several genes that encode antioxidant proteins and enzymes [12]. Pancreatic beta cells are particularly sensitive to free radicals, which induce a decrease in insulin secretion [13] because of their low level of antioxidant enzymes such as superoxide dismutase (SOD), catalase (CAT), and glutathione peroxidase (GPx) [14,15]. However, the balance between ROS and the antioxidant defense system is essential to maintain homeostasis; if this equilibrium is damaged in favor of the pro-oxidant proteins, pathological oxidative stress occurs [16]. Regarding this sensitivity and metabolism impairment, the research of agents that may modulate or prevent oxidative stress is essential for the future development of therapeutic strategies against diabetes. In this regard, the use of plants as a source of natural antioxidants, which may prevent and protect against oxidative stress in biological systems is booming [17].

Medicinal plants are the richest sources of biologically active substances and traditional systems of medicine for synthetic drugs [18]. Phytochemical screening is very important in detecting new sources of therapeutically and industrially useful compounds such as flavonoids, alkaloids, phenolic compounds, tannins, and terpenoids [19]. These compounds can be extracted from any part of the plant, such as bark, leaves, flowers, and seeds [20].

Jojoba (*Simmondsia chinensis* (Link) C. K. Schneid., family Simmondsiaceae), (Syn. *Buxus chinensis* Link; *Simmondsia californica* Nutt.) is a coffee berry, wild hazel, and goat nut [21]. It is dioecious plant, typical of south-western US and north-western Mexico, growing in desert and semi-desert areas [21]. The oil makes up approximately 50% of the jojoba seed by weight, and simmondsin is one of the principal component [22].

The meal of jojoba seeds is used as livestock feed ingredient [23]. It is rich in proteins (29–30%), cyanogenic glycosides, and simmondsin and its derivatives [24,25,26,27]. The meal also includes some carbohydrates such as 4-β-galactobiose, 4-β-galactotriose, 1D-2-O-α-d-galactopyranosyl-chiro-inositol, d-pinitol, sucrose, 2-α-d-galactopyranosyl-d-pinitol, and 5-α-d-galactopyranosyl-d-pinitol [28,29]. This plant, known to Native Americans for its medicinal purposes, is used as a remedy for obesity, cancer, wounds, and throat warts [30,31].

Jojoba seed oil (liquid wax ester) has many medicinal benefits such as anti-inflammatory [32], wound healing, skin disorder healing [31], antioxidant [33], and lubricant properties [34]. In addition, from jojoba pericarp, few flavonoids such as quercetin-3,3′-dimethyl ether, isokkaempferide, and quercetin 3-methyl ether [35] have been isolated.

Simmondsins, the major molecules present in jojoba, are known as antifungal, antifeedant, and insecticidal [36]. However, the impact of the pure molecule simmondsin has not been described yet, and its effect has not been compared to that of the complete seed extract containing phenolic compounds.

The aims of the present study were to evaluate the antioxidant properties of the aqueous extract of jojoba seeds on oxidative stress induced by hyperglycemia in RINm5f beta cell lines and to compare its effect to that of a pure simmondsin extract known to bring the beneficial properties of jojoba.

## 2. Materials and Methods

### 2.1. Collection and Extraction of Plant Material

The seeds were collected in July 2013 from Meknassy, Sidi Bouzid, Tunisia (latitude: 34°38′ N, longitude: 9°37′ E, altitude: 223 m above sea level (a.s.l.)). The seeds were authenticated by Fakhreddine Khaskhoussi (agricultural engineer) working in jojoba fields.

Aqueous extraction was performed by magnetically stirring of 100 g of jojoba seeds for 2 h with 500 mL of water at 90 °C in a round-bottomed flask provided with a reflux condenser [27]. The supernatant was filtered with filter paper to separate the jojoba oil, and the residue was added with 30 mL of methanol. In order to eliminate insoluble products, an extra filtration was performed. The solvent was evaporated by a rotary evaporator (Buchi, Rungis, France), and the brown residue obtained was stored at 4 °C for further experiments.

Synthetic Simmondsin was purchased from Boc Sciences (Shirley, NY, USA).

### 2.2. Identification and Quantification of Phenolic Compounds by Reversed-Phase High-Performance Liquid Chromatography (RP-HPLC)

Separation and analysis of the phenolic compounds from the aqueous jojoba seed extract was carried out by RP-HPLC using the Dionex Ultimate 3000 analytical equipment (Lab X, Midland, ON, Canada) with an ultra violet (UV)-visible detector (barrier detector of Diodes 3000RS) and with an ACE C18-PFP column (250 × 4.6 mm, 5 μm) (Advanced Chromatography Technologies, Aberdeen, Scotland) at ambient temperature (37 °C) and at a maintained flow rate of 1 mL/min. The mobile phase consisted of water with 0.1% formic acid (solvent A) and acetonitrile (solvent B). The program gradient was as follows: 97% A/3% B for 0–35 min, 79.5% A/20.5% B for 35–45 min, and 72% A/3% B for 66–76 min. The volume injected was 20 μL, and the chromatographic profiles were examined at 280 nm. The peaks of the phenolic compounds were identified according to their retention time by co-injection of pure controls of phenolic acids and flavonoids under the same experimental conditions.

### 2.3. Cell Culture and Treatment

A rat insulinoma cell line (RINm5f beta) was purchased from the American Type Culture Collection (ATCC, Manassas, VA, USA). Cells from passages 30–43 were used. The cells were grown at 37 °C under a humidified 5% CO_2_ atmosphere in Roswell Park Memorial Institute (RPMI-1640, Sigma, St. Louis, MO, USA) medium supplemented with 10% fetal bovine serum (FBS, Sigma-Aldrich, St. Louis, MO, USA) and 1% antibiotic-antimycotic (ABAM, Gibco-Invitrogen, Grand Island, NY, USA). RINm5f beta cells were trypsinized using 0.05% trypsin ethylene diamine tetraacetic acid (EDTA, Sigma-Aldrich, St. Louis, MO, USA) at 80% confluence and loaded in a 96-well plate (Dutscher, Issy-les-Moulineaux, France) or 6-well plate (Dutscher) at a concentration of 10^5^ cells/200 µL and 10^6^ cells/mL, respectively. The medium was changed every two days. Twelve hours before starting the treatment, the cells were starved and incubated with different concentrations of plant extracts (20, 50, 80, 100, 150, 200, and 300 µg/mL for the aqueous jojoba seed extract, and 10, 20, 40, 80, and 150 µg/mL for simmondsin) for 24 h to study the toxicity on RINm5F beta cell lines (Figure S1A). To validate the antioxidant properties of the extracts, cells were incubated with selected concentrations of plant extracts for 24 h before inducing an oxidative stress by 250 mM of fructose for 24 h (Figure S1B).

### 2.4. Cell Viability and Functionality

Viability of RINm5f beta cells was determined by measuring the mitochondrial activity with the Cell Titer 96^®^ AQueous One Solution Cell Proliferation Assay (Promega, Madison, WI, USA). After treatment, 20 µL of 3-(4,5-Dimethylthiazol-2-yl)-2,5-diphenyltetrazolium bromide (MTT) and 100 µL of culture medium was added.

Cells were incubated for 2 h at 37 °C in 5% CO_2_, and the absorbance was measured at 490 nm with the Meterech 960 microplate reader (Metertech Inc., Taipei, Taiwan). The color development was proportional to the number of viable cells. The results are presented in percentage (%) viability with respect to the negative control (cells treated in the same condition but only with solvent).

The functionality of RINm5f beta cells was determined by measuring static insulin release. Briefly, the supernatant of the cells after each treatment was collected and stored at −80 °C. Insulin release was measured using the Rat Insulin ELISA kit (Mercodia, Uppsala, Sweden) and expressed in mg/mL.

### 2.5. ROS Production

Cells were seeded in 96-well treated black microplates (Greiner Bio-One, Les Ulis, France) at 10^5^ cells/well. Cells were treated with 2′,7′-Dichlorodihydrofluorescein diacetate (DCFH-DA, 60 µM, Sigma-Aldrich, Steinheim, Germany) and different concentrations of the jojoba extracts dissolved in RPMI-1640 medium. After rinsing the cells with Hanks’ balanced salt solution (HBSS) buffer, they were exposed to oxidative stress with fructose (250 mM). Trolox (Sigma-Aldrich, St. Louis, MO, USA) was used as a reference antioxidant.

Since the initiation of the oxidative stress, the fluorescence signal (excitation wavelength (λ_Ex_) = 485 nm; emission wavelength (λ_Em_) = 538 nm) was recorded for 100 min. The CAA (cellular antioxidant activity) value was calculated as Equation (1).

(1) $$\mathrm{CAA}=1-\left(1-\frac{\int \mathrm{sample}-\int \mathrm{Blank}}{\int \mathrm{Control}-\int \mathrm{Blank}}\right)$$

### 2.6. Determination of Caspase-3 Activation

Caspase-3 activation in the protein lysate was determined by the Human Active Caspase-3 kit (Quantikine^®^ ELISA, Bio-Techne, Minneapolis, MN, USA) by using Elisa plaques. Results were expressed as percentage (%) relative to the untreated cells.

### 2.7. Protein Extraction and Western Blotting Analyses

To study the effects of jojoba extracts on the expression levels of proteins of interest, the cells were rinsed with 500 µL phosphate-buffered saline (PBS) and lysed with mammalian buffer (N-PERTM, Thermo Fisher Scientific, Illkirch-Graffestaden, France) (200 µL) and 10 µL of a cocktail of protease inhibitors (Roche Diagnostics, Meylan, France). Protein concentration was quantified using the Bradford test [37], and bovine serum albumin (BSA, Sigma Aldrich, Saint-Quentin Fallavier, France) was used as a protein standard.

For western blotting, 10 µg of protein was separated in a 4–12% Bis-Tris Criterion XT precast gel (Bio-Rad, Marne-La-Coquette, France) and transferred to poly (vinylidene) fluoride (PVDF) membranes (Millipore, Molsheim, France). After blocking with 5% (*w*/*v*) BSA in 0.1% (*v*/*v*) PBS-Tween 20 and washing in Tris phosphate buffered saline (TPBS), the membranes were incubated overnight at 4 °C with anti-p22phox (1/500) (Thermo Fisher, Invitrogen, Paris, France)., anti-Nrf2 (Thermo Fisher) (1/500), anti-Mn-superoxide dismutase (MnSOD) (1/1000, Sigma-Aldrich), and anti-CAT (1/1000, Sigma-Aldrich) antibodies. The membranes were incubated with a secondary antibody coupled to horseradish peroxidase (HRP). Raw quantification of protein levels was performed by comparison with β-actin, a reference protein, using ImageJ software (NIH Laboratories, Bethesda, MD, USA). Results were expressed as percentage (%) relative to the untreated cells.

### 2.8. Antioxidant Enzyme Activities

After removing the supernatant, cells were washed with PBS, pH 7.4, resuspended in 200 µL of ice-cold Assay buffer (N-PERTM, Thermo scientific, Waltham, MA, USA), and homogenized quickly by pipetting. Samples were centrifuged for 15 min at 4 °C at 15,000× *g*. Supernatants were collected and transferred to clean tubes.

To measure SOD and CAT activities, we used the Superoxide Dismutase Activity Assay kit (colorimetric, Abcam, Paris, France) and Catalase Activity Assay kit (fluorometric, Abcam), respectively, according to the manufacturer’s instructions. Results were expressed as percentage (%) of inhibition rate.

### 2.9. Statistical Analysis

Statistical analysis was performed by one-way analysis of variance (ANOVA) and by Fischer’s test using the Statistica software (StatSoft, Tulsa, OK, USA).

All data were given as mean ± standard error of the mean (SEM). Differences were considered statistically significant at *p*-value < 0.05 (*, $), *p*-value < 0.01 (**, $$), or *p*-value < 0,001 (***, $$$).

## 3. Results

### 3.1. Characterization of the Phenolic Compounds from the Aqueous Jojoba Seed Extract

Phytochemical profiling of the aqueous seed extract of *S. chinensis* using RP-HPLC revealed the presence of 27 constituents.

Table 1 shows the list of compounds identified through RP-HPLC experiments along with their retention time (RT). The percentage of total phenolics detected was 87.95%. Different classes of polyphenols have been identified: hydroxybenzoic acid (45.09%), flavonoids (32.59%), anthocyanins (1.56%), and hydroxycinnamic acids (8.71%) (Table 1 and Figure S2).

### 3.2. Cell Viability

**Figure 1:**
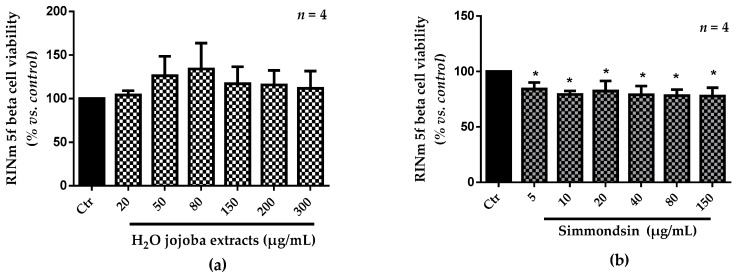
Effect of the aqueous jojoba seed extracts (**a**) and simmondsin (**b**) on the viability of RINm5f beta cells at different concentrations. Each value represents the mean ± standard error of the mean (SEM) of three independent experiments in triplicate. * *p* < 0.05, compared to untreated cells. Ctr: control.

The toxicity of the aqueous jojoba seed extract (H_2_O extract) and simmondsin is presented in Figure 1. Cell viability was preserved using up to 300 µg/mL H_2_O extract. However, a significant reduction in cell viability was noticed after treatment with pure simmondsin (*p* < 0.05). This reduction was not dose-dependent.

**Figure 2:**
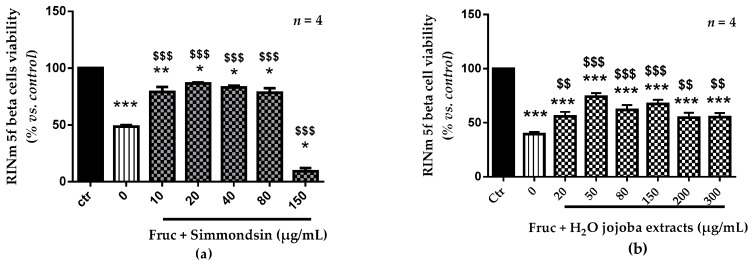
Effect of pure simmondsin (**a**) and the aqueous extract of jojoba seeds (**b**) on viability of RINm5f beta cells in the presence of fructose (250 mM). Each value represents the mean ± standard error of the mean (SEM) of three independent experiments in triplicate. * *p* < 0.05, ** *p* < 0.01, *** *p* < 0.001, compared to untreated cells, * *p* < 0.05, ^$$^ *p* < 0.01, ^$$$^ *p* < 0.001, compared to fructose-treated cells. Fruc: fructose.

After oxidative stress induced by 250 mM of fructose (Figure 2), we observed a decrease in the viability of RINm5F beta cells (48.5% ± 1.3). The pre-treatments with simmondsin pure extract showed a significant increase in cell viability from 45.5% to 86.6 (20 µg/mL, *p* < 0.001) and to 83.2% (40 µg/mL, *p* < 0.001). The same results were obtained with jojoba seed extract, with an increase in cell viability from 39.5% to 74.0% (the half maximal inhibitory concentration at 50 µg/mL).

The choice of concentration for the next experiments is a compromise between the toxicity of the extracts and their biological effectiveness following a fructose-induced oxidative stress. We selected 150 mg/mL for the extracts (more effective after inducing oxidative stress and without toxicity) and 40 mg/mL for simmondsin which has comparable efficiency to 20 μg/mL.

### 3.3. Effect of Jojoba Extracts on Static Insulin Release

**Figure 3:**
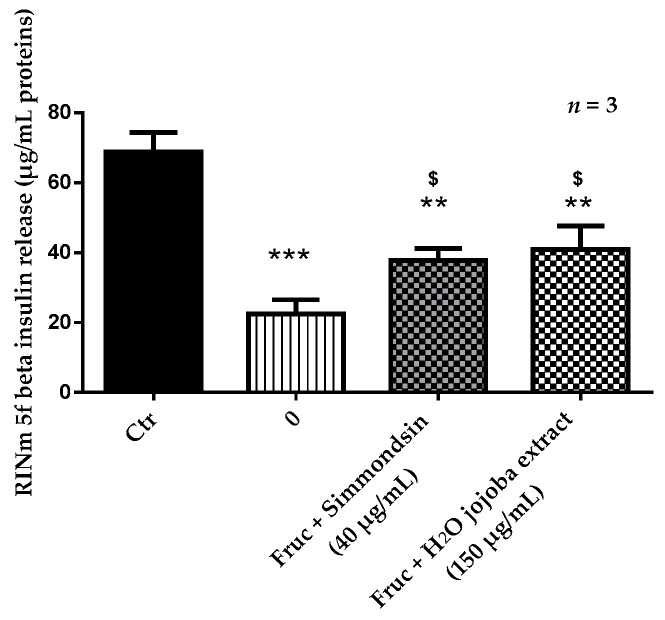
Effect of the aqueous jojoba seed extract and pure simmondsin on RINm5f beta cell static insulin release in the presence of fructose. Each value represents the mean ± standard error of the mean (SEM) of three independent experiments in triplicate. ** *p* < 0.01, *** *p* < 0.001, compared to untreated cells, ^$^ *p* < 0.05, compared to fructose-treated cells.

The static insulin release of RINm5f beta cells was significantly reduced from 68.7 ± 2.23 to 22.46 ± 2.36 (*p* < 0.001) in the presence of fructose 250 mM alone. Simmondsin significantly increased insulin release (37.77 ± 2.00, *p* < 0.05) compared to fructose alone, even though this secretion always appeared significantly reduced compared to the control (*p* < 0.01). The same results were obtained with the aqueous jojoba seed extract (Figure 3).

### 3.4. Jojoba Extracts Prevent Caspase-3 Activation and Oxidative Stress Induced by Fructose

**Figure 4:**
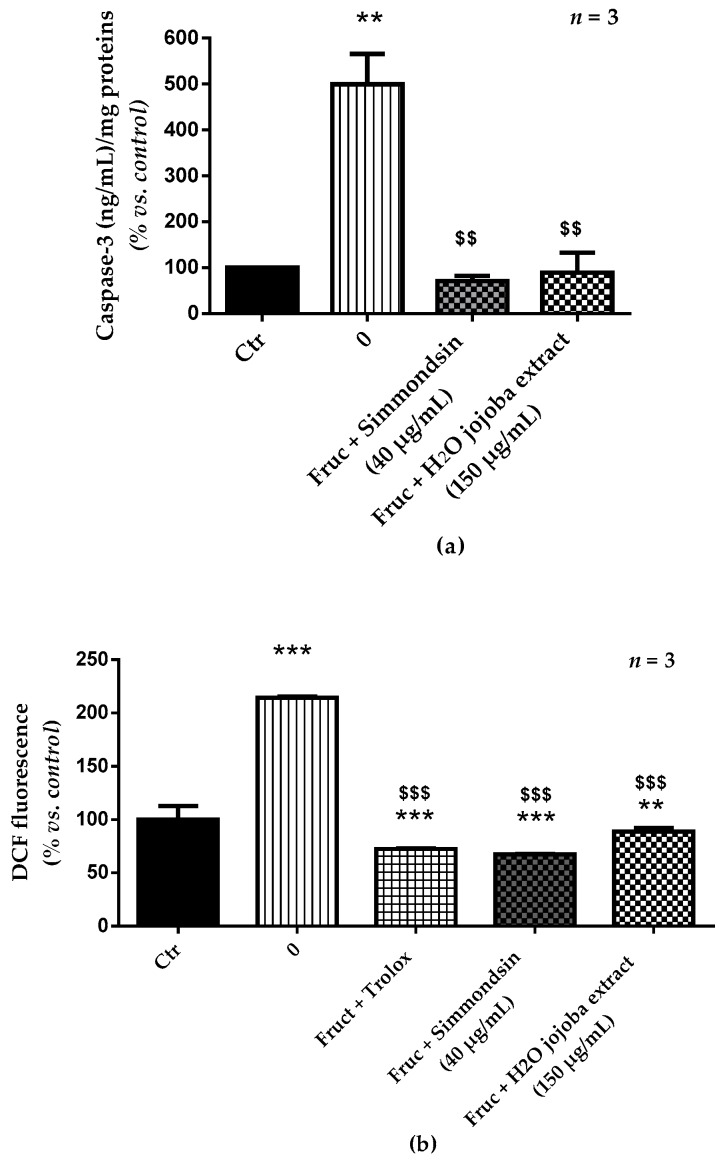
Effect of the aqueous jojoba seed extract and pure simmondsin on caspase-3 activation (**a**) and oxidative stress (**b**). Each value represents the mean ± standard error of the mean (SEM) of three independent experiments in triplicate. ** *p* < 0.01, *** *p* < 0.001, compared to untreated cells, ^$$$^ *p* < 0.001, compared to fructose-treated cells.

As shown in Figure 4, the caspase-3 activation significantly increased (100% ± 0.40% vs. 499.50% ± 66.24%, *p* < 0.01) after fructose (250 mM) treatment during 24 h compared to that in untreated cells. This increase was correlated with a significant increase in intracellular ROS generation (from 100% ± 12.80% to 214.27% ± 1.29%, *p* < 0.001). Simmondsin pure extract reduced significantly caspase-3 activation and ROS production with levels comparable to the normal state (*p* < 0.001). Similar results were obtained with the aqueous jojoba seed extract.

### 3.5. Enzymatic Antioxidant Defense Status and Oxidative Stress Damage

**Figure 5:**
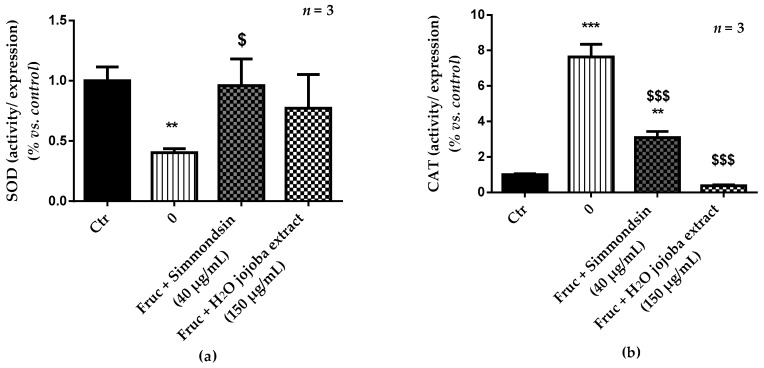
Effect of the aqueous jojoba seed extract and pure simmondsin on superoxide dismutase (SOD) (**a**) and catalase (CAT) (**b**) ratio (activity/expression). Each value represents the mean ± standard error of the mean (SEM) of three independent experiments in triplicate. * *p* < 0.05, ** *p* < 0.01, *** *p* < 0.001, compared to untreated cells, ^$^ *p* < 0.05, ^$$$^ *p* < 0.001, compared to fructose-treated cells.

Exposure to high fructose concentration induced a significant decrease (0.40 ± 0.33, *p* < 0.01) of SOD ratio (activity/expression) (Figure 5a) confirmed by its overexpression (Figure S3). This reduction was prevented by simmondsin and jojoba seed extract (0.96 ± 0.22 and 0.77 ± 0.28, respectively), with a decrease in its expression. In contrast, fructose induced a significant increase (7.63 ± 0.72, *p* < 0.001) in CAT ratio (activity/expression) (Figure 5b), indicated by an increase in its activity and a decrease in its expression. This increase was partially reduced by the simmondsin extract (3.10 ± 0.34, *p* < 0.01 vs. control), but it was completely inhibited by jojoba seed extract (0.38 ± 0.04, no significant difference vs. control).

### 3.6. Effect of Jojoba Extracts on Pro-Oxidant Signaling Pathway

**Figure 6:**
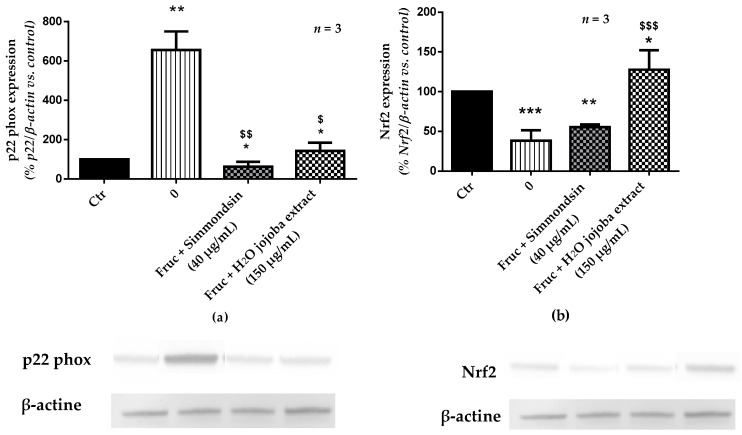
Effect of the aqueous jojoba seed extract and simmondsin on p22phox (**a**) and nuclear factor (erythroid-derived 2)-like 2 (Nrf2) expression (**b**). Each value represents the mean ± standard error of the mean (SEM) of three independent experiments in triplicate. * *p* < 0.05, ** *p* < 0.01, *** *p* < 0.001, compared to untreated cells, ^$^ *p* < 0.05, ^$$^ *p* < 0.01, ^$$$^ *p* < 0.001, compared to fructose-treated cells.

Exposure of RINm5F beta cells to high concentration of fructose (250 mM) for 24 h induced a significant increase (655.72% ± 94.82%, *p* < 0.01) in the expression of the p22phox subunit of NADPH oxidase (Figure 6a). The incubation of the cells with simmondsin significantly reduced this expression (62.57% ± 24.15%, *p* < 0.01). This reduction was less significant with jojoba seed extract (142.28 ± 41.80, *p* < 0.05).

Surprisingly, in the presence of fructose, Nfr2 expression was significantly inhibited (38.24% ± 7.69%, *p* < 0.001) (Figure 6b). However, addition of the simmondsin extract had no impact on Nrf2 levels (55.45% ± 1.95%). In contrast, addition of jojoba seed extract significantly increased Nfr2 expression (127.59 ± 14.15, *p* < 0.001 compared to fructose). Moreover, this overexpression of Nrf2 was also observed compared to control conditions (*p* < 0.05).

## 4. Discussion

In this study, we aimed to investigate the effect of the aqueous extract of jojoba seeds on hyperglycemia-induced oxidative stress in RINm5f beta cells and to compare this effect to the simmondsin, a pure extract of Jojoba.

Our results showed a protective effect of extracts against hyperglycemia-induced oxidative stress through the modulation of RINm5f beta cell cytotoxicity, generation of ROS, insulin release, caspase-3 activation, pro-oxidant and antioxidant defense, and status of the cells. In fact, the effect of the aqueous extract of jojoba seeds was comparable to that of simmondsin pure extract, even though the signaling pathway was different.

The RP-HPLC analysis of the aqueous jojoba seed extract showed the presence of 27 molecules belonging to the polyphenol family belonging to 4 classes: hydroxybenzoic acids, flavonoids, anthocyanins, and hydroxycinnamic acids. Bellirou et al. [27] used another type of HPLC to determine the composition of this extract, and they showed the presence of simmondsin and its seven derivatives. The isolation of simmondsin was first described by Elliger et al. [38]. Many methods for quantitative analysis of simmondsin and related substances in jojoba seeds have been described [23,39,40]. Limitations in the detection of jojoba seed constituents are associated with these methods. In fact, simmondsin, demethyl simmondsin, simmondsin ferulate, and didemethyl simmondsin have either positional or geometric isomers [40]. Thus, the degree by which these isomers are separated by various procedures and which isomeric peaks are measured lead to differences in analysis results from different laboratories.

We demonstrated that the aqueous extract of jojoba seeds was not toxic for RINm5f beta cell lines, but a low toxicity was observed for pure simmondsin extract, which was not dose-dependent. The toxicity and the non-toxicity of simmondsin in vivo were discussed by several authors. Oral administration of simmondsin in starved rats did not lead to adverse effects, even if chronic oral administration reduced the weight of rats, and anovulatory ovaries were observed in female rats [41]. Furthermore, a long-term low-dose administration of simmondsin in rats showed no toxic effects [42], whereas a high dose of simmondsin (5%) in the diet caused mortality in rats [43].

Many studies have been performed in pancreatic beta cells, and the key role of flavonoids as cell cycle regulators in many cell types suggests that they may have effects on beta cell proliferation, as demonstrated by our study with jojoba seed extract. In fact, numerous flavonoids modulate cell proliferation. For example, monomers such as quercetin [44], procyanidins [45,46], gallic acid [47], and other flavonoids such as genistein [48] modulate cell proliferation in many cell types, including osteoblasts [44], fibroblasts [48], and several carcinoma cell lines [46]. Auberval et al. [49] tested epigallocatechin gallate (EGCG) in RINm5f beta cells and showed that EGCG induced an increase in cell viability at concentrations of 500 μg/mL and 1000 μg/mL. In contrast, many studies established that polyphenols were capable of inhibiting cell proliferation by arresting cell cycle and apoptosis in several solid tumor cell lines [50,51,52,53,54].

However, in the presence of high concentrations of fructose, we showed a decrease in beta cell proliferation and insulin release, and an increase in ROS production associated with a modulation of antioxidant/pro-oxidant enzymes. This was associated with a strong increase in apoptosis, attested by caspase-3 activation. Fructose is the sugar naturally present in fruits, and it is used instead of glucose as a source of carbohydrates [55]. Fructose is absorbed and rapidly converted into glucose by liver cells as well as other tissues [56]. Deleterious effects were described both for high-fructose and high-glucose concentrations [57,58]. In addition, the consumption of a high amount of glucose in the diet increases the risk of obesity [59,60], dyslipidemia [61], heart disease [62], resistance to insulin [63], oxidative stress [64], and apoptosis and caspase-3 activation [65].

Pre-treatment with simmondsin or the aqueous jojoba seed extract preserved cell viability with a decrease in ROS production, apoptosis, and maintenance of insulin release. The aqueous jojoba seed extract contains simmondsin, but its protective effect against oxidative stress has not been shown yet.

In addition, the aqueous jojoba seed extract and simmondsin remarkably decreased ROS generation, caspase-3 activation, and antioxidant enzyme levels, and increased insulin release. Taken together, the results of our study showed that simmondsin alone reduced oxidative stress induced by fructose, but the synergistic effect of other molecules of different phenolic classes remains unknown.

This beneficial effect of the aqueous jojoba seed extract may be due to the presence of compounds such as quercetin, quercetin 3-glucoside, catechin, and caffeic acid. Lapidot et al. [66] showed the protective effect of these components on cell proliferation after oxidative stress. In addition, the administration of quercetin inhibited oxidative stress in the liver of streptozotocin-induced hyperglyciemia in rats [67].

The antioxidant effect of the aqueous jojoba seed extract may also be due to the presence of gallic acid, an endogenous plant phenol, abundantly present in tea, grapes, berries, fruits as well as wine [68,69]. Kim et al. [70] reported the potent free radical scavenging and antioxidant properties of gallic acid. The presence of rutin in the aqueous jojoba seed extract may also prevent fructose-induced oxidative stress and production of ROS by scavenging hydrogen peroxide (H_2_O_2_). However, the direct antioxidant effect of rutin cannot explain the long-lasting effect on human health, given its relatively short half-life.

Simmondsin alone also decreased the production of ROS induced by high concentration of fructose. No scientific reports on the effects of simmondsin on oxidative stress and its mechanisms of action are available. However, our data suggest that simmondsin is a scavenger on H_2_O_2_ molecules resulting from fructose-induced oxidative stress.

We confirmed that oxidative stress induced by fructose decreased insulin secretion [71] and demonstrated that the aqueous jojoba seed extract and simmondsin helped beta cells to preserve insulin secretion ability under long-term fructose exposure (24 h). For the aqueous jojoba seed extract, the effect may be the consequence of the presence of polyphenols such as quercetin, quercetin gallate, and rutin. Cai and Lin [71] showed that EGCG and rutin enabled pancreatic beta cells to sustain the adaptation to hyperglycemia and attenuate the glucotoxicity produced by high concentrations of glucose. In addition, EGCG and rutin attenuated the glucotoxic effects through activating the insulin receptor substrate 2 (IRS2) and adenosine monophosphate-activated protein kinase (AMPK) signaling pathways. The enhancement of insulin signaling to increase the active tyrosine phosphorylation signaling and to decrease the negative serine phosphorylation signaling preserves the insulin sensitivity and facilitates the downstream signaling, including serine/threonine kinase (Akt), forkhead-O transcription factor 1 (FoxO1), and pancreas-duodenum homeobox-1 (PDX-1). Another mechanism of action that explains the increase in insulin secretion by the polyphenols may be the direct activation of l-type calcium channels by quercetin [72]. The presence of kaempferol in the aqueous extract of jojoba seeds may also help the cells to secrete insulin. According to Zhang et al. [73], exposure of beta cells to high glucose reduced cyclic AMP (AMPc) levels, which were partially restored by co-incubation with kaempferol.

The precise mechanism by which simmondsin causes an increase in insulin has not been studied yet. We speculated that the increase in the passage of the glucose to pancreatic cells through glucose transporter 2 (GLUT2) or activation of the signaling neighbors of IRS2 and AMPc may contribute to the increase in insulin.

Caspases are key mediators of cell death, and caspase-3 is an executioner caspase responsible for the death program in many cells in response to oxidative stress. Our data indicated that the exposure of RINm5f beta cells to a high concentration of fructose damaged beta cells, leading to the increase in caspase-3 levels. The pre-treatment with simmondsin and the aqueous jojoba seed extract may protect pancreatic beta cells from high concentrations of fructose that induce apoptosis and cell dysfunction. The protective effect of the aqueous jojoba seed extract may be attributed to the richness in polyphenols such as kaempferol. It protects against the increase of caspase-3 levels by decreasing the apoptosis, allowing the generation of the AMPc and regulation of the expression of Akt and B-cell lymphoma 2 (Bcl-2) [74]. In addition, the decrease in caspase-3 activation may be due to the presence of rutin, which increases caspase-3 expression in rats by inhibiting the anti-apoptotic protein Bcl-2 [75]. Several recent studies have also demonstrated that apigenin has antiapoptotic properties in in vitro and in vivo models [76,77]

Oxidative stress results from an imbalance between ROS formation and antioxidant defenses. Antioxidant enzymes such as SOD and CAT are the primary cellular antioxidant defenses [78]. SOD converts superoxide radicals (O_2_●^−^) to H_2_O_2_, while CAT converts H_2_O_2_ to water (H_2_O) and oxygen molecule (O_2_) in the peroxisomes [79]. The stability and capacity of the antioxidant defense against ROS during diabetes plays a key role in the outcomes of complications caused by ROS [80].

Rodriguez et al. [81] demonstrated an increase in SOD and CAT activation following the increase in ROS generation caused by oxidative stress, indicating their role in protection against oxidative stress. In fact, an increase in antioxidant enzymes after fructose-induced oxidative stress has been reported [82,83,84]. The increased levels of antioxidant enzymes stimulated cellular capacity to scavenge free radicals, limiting the damage caused by ROS [83], even though the increased expression observed in our study was not sufficient to counteract the oxidative stress induced by fructose. However, antioxidant enzymes levels ameliorated in the presence of the aqueous jojoba seed extract and simmondsin because of an increased expression of CAT, as described in previous studies [85]. Rutin and quercetin in the aqueous jojoba seed extract may explain this activation [86,87].

Concerning the pro-oxidant signaling pathway, an activation of the pro-oxidative agents involving the p22phox subunit was demonstrated during the fructose-induced oxidative stress. p22phox is a protein also known as human neutrophil cytochrome b light chain. It is an essential protein involved in the NADPH-oxidase bound to the membrane [88]. NADPH oxidase uses an electron donor, NADPH, or reduced nicotinamide adenine dinucleotide (NADH) to reduce the electron oxygen under oxidative stress to donate O_2_●^−^, which is a ROS [89]. The aqueous extract of jojoba seeds and simmondsin are able to substitute part of the antioxidant enzymatic machinery, presumably by trapping the free radicals produced by the hyperglycemic stress and avoiding the increased expression of p22phox, reducing the production of ROS. The mechanism of action of the aqueous jojoba seed extract and simmondsin blocks ROS production through p22phox or by influencing the expression of cellular antioxidant enzymes.

Nrf2 has a beneficial role in regulating NADPH generation and consumption [90]. Nrf2 controls the basal and induced expression of an array of antioxidant response element-dependent genes, regulating the physiological and pathophysiological outcomes of oxidant exposure. Its stimulation is a potential target for preventing oxidative stress [91] and diabetes complications [92]. It adapts the expression of several genes such as those that control antioxidant enzymes [93]. Our results showed that oxidative stress induced by fructose decreased Nrf2 expression, but pre-treatment of RINm5f beta cells with simmondsin and especially the aqueous extract of jojoba seeds increased the expression of this factor.

In our study, only the jojoba seed extract was able to activate Nrf2 after oxidative stress. This activation may be explained by the presence of apigenin, rutin, quercetin, and kaempferol in the aqueous jojoba seed extract, which activate Nrf2 by promoting Nrf2 translocation into the nucleus. 

## 5. Conclusions

In conclusion, according to our results on the prevention of oxidative stress, the aqueous extract of jojoba seeds has the same effect of simmondsin, suggesting that the use of pure simmondsin as a drug to prevent oxidative stress induced by fructose may be a promising strategy against oxidative stress. However, the molecular signaling pathway associated with the aqueous extract of jojoba seeds containing phenolic compounds but without simmondsin has a more complete and synergistic effect because of its richness and its diversity in antioxidant molecules. Further studies on the effect of jojoba seeds on the prevention of diabetes and its complications are necessary.

## Figures and Tables

**Table 1:** Phenolic compounds detected in the aqueous jojoba seed extract by using reversed-phase high-performance liquid chromatography (RP-HPLC).

Peak No.	RT	%	Identified Compound
1	3.2	36.60	gallic acid
2	4	4.46	3,5-dihydroxybenzoic acid
3	20.6	1.12	*p*-hydroxybenzoic acid
4	23.8	1.12	caffeic acid hexoside
7	27.7	1.34	syringic acid
5	24.4	1.79	catechin
8	28.7	2.23	kaempferol 3-glucoside
10	29.8	1.12	apigenin 7-rutinoside
12	31.7	1.34	quercetin-3,4-diglucoside
15	34.3	1.12	quercetin trisaccharide
19	38.1	7.14	rutin
22	45.6	5.58	epicatechin gallate
23	50.5	1.12	quercetin 3-glucoside
24	59.1	4.46	quercetin
25	59.6	6.7	isorhamnetin-3-glucoside
9	29.2	1.56	cyanidin-3-rutinoside
6	25	1.56	caffeic acid
16	35.7	1.12	*p*-coumaric acid
20	41.5	3.79	ferulic acid
21	42.8	2.23	sinapic acid
11	31.3	0.89	nd
13	33.3	1.34	nd
14	33.9	0.89	nd
17	36.5	3.79	nd
18	37.5	1 .12	nd
26	61	2.90	nd
27	62	1.56	nd

nd: not determined, RT: retention time.

## Supplementary Materials

The following are available online at http://www.mdpi.com/2072-6643/10/3/384/s1. Figure S1. Schematic representation of (A) the toxicity test (B) the toxicity test after fructose stress, Figure S2. Chromatogram of aqueous 2 jojoba seeds extract, Figure S3. Effect of simmondsin and aqueous extract on fructose induced-oxidative stress.
