# Supplementary material for: Beneficial Effect of Jojoba Seed Extracts on Hyperglycemia-Induced Oxidative Stress in RINm5f Beta Cells
Source: Nutrients. 2018 Mar 20;10(3):384. doi: 10.3390/nu10030384 (PMC5872802; doi:10.3390/nu10030384)
Supplement: Supplementary file 1 [file nutrients-10-00384-s001.pdf]

Supplementary Materials

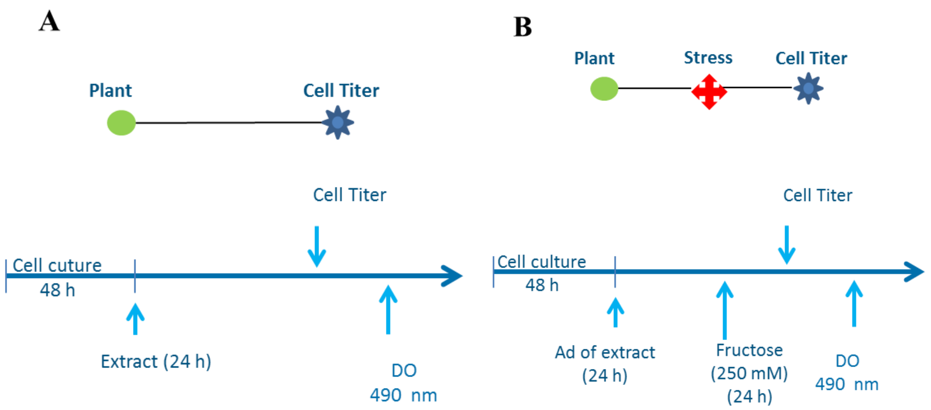

Figure S1. Schematic representation of (**A**) the toxicity test (**B**) the toxicity test after fructose stress.

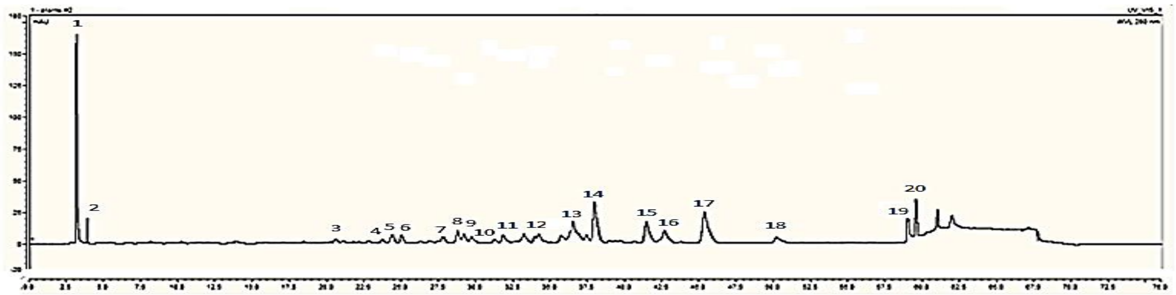

Figure S2. Chromatogram of aqueous 2 jojoba seeds extract.

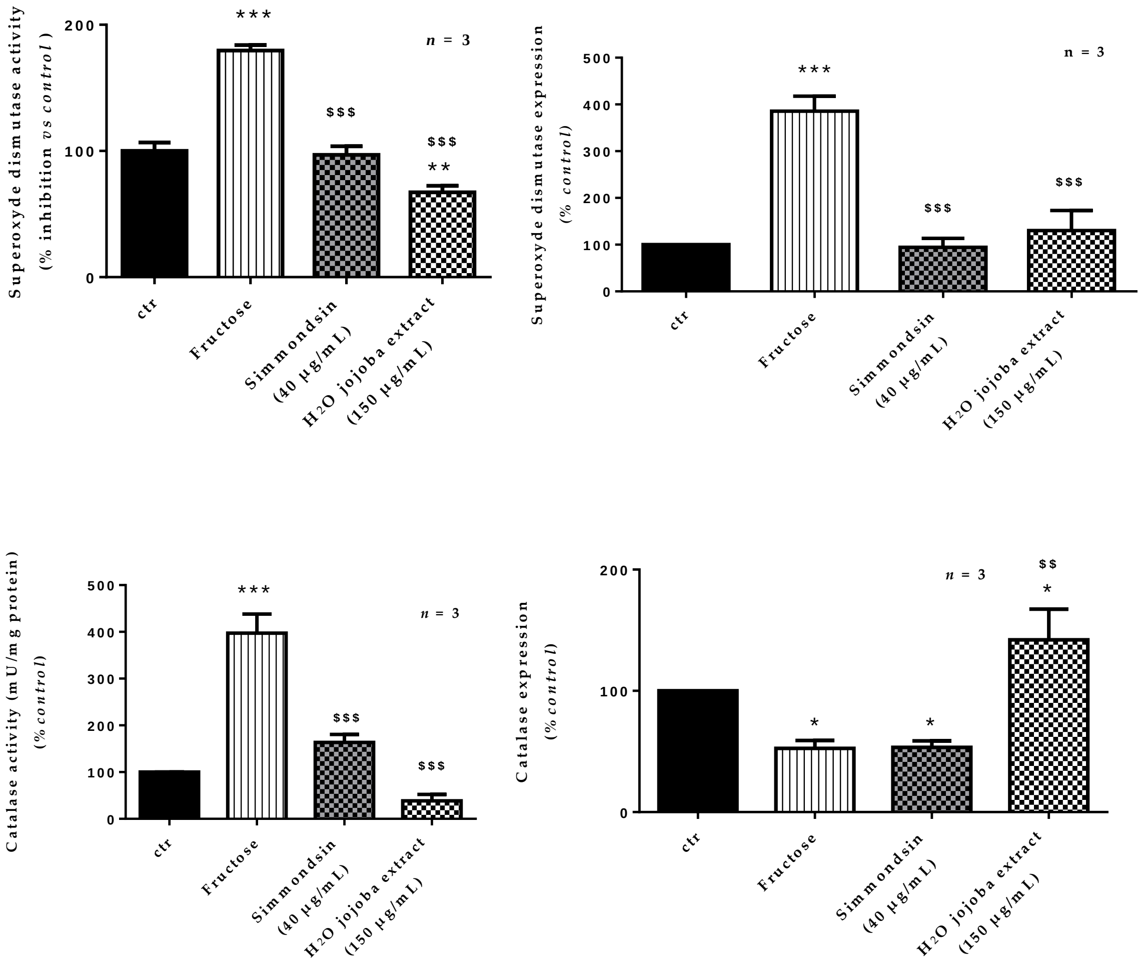

Figure S3. Effect of simmondsin and aqueous extract on fructose induced-oxidative stress. Each value represents the mean ± standard error of the mean (SEM) of three independent experiments in triplicate. * $p < 0.05$, ** $p < 0.01$, *** $p < 0.001$ as compared to untreated cells, \$ $p < 0.05$, \$\$ $p < 0.01$, \$\$\$ $p < 0.001$ as compared to Fructose or insulin treated cells. $n = 6$.
